# Fatigue in Medical Residents Leads to Reactivation of Herpes Virus Latency

**DOI:** 10.1155/2011/571340

**Published:** 2011-12-20

**Authors:** Peter N. Uchakin, David C. Parish, Francis C. Dane, Olga N. Uchakina, Allison P. Scheetz, Neal K. Agarwal, Betsy E. Smith

**Affiliations:** ^1^Department of Internal Medicine, Mercer University School of Medicine, 1550 College Street, Macon, GA 31207, USA; ^2^Department of Community Medicine, Mercer University School of Medicine, Macon, GA 31207, USA; ^3^Department of Arts & Sciences, Jefferson College of Health Sciences, Roanoke, VA 24013, USA; ^4^Division of Basic Medical Sciences, Mercer University School of Medicine, Macon, GA 31207, USA; ^5^Department of Emergency Medicine, Long Island Jewish Medical Center, NSLIJ Health System, Albert Einstein Medical School, Bronx, NY 11040, USA

## Abstract

The main objective of this study was to detect fatigue-induced clinical symptoms of immune suppression in medical residents. Samples were collected from the subjects at *rest*, following the first night (*low-stress*), and the last night (*high-stress*) of night float. Computerized reaction tests, Epworth Sleepiness Scale, and Wellness Profile questionnaires were used to quantify fatigue level. DNA of human herpes viruses HSV-1, VZV, EBV, as well as cortisol and melatonin concentrations, were measured in saliva. Residents at the *high-stress* interval reported being sleepier compared to the *rest* interval. EBV DNA level increased significantly at both *stress* intervals, while VZV DNA level increased only at *low-stress*. DNA levels of HSV-1 decreased at *low-stress* but increased at *high-stress*. Combined assessment of the viral DNA showed significant effect of stress on herpes virus reactivation at both *stress* intervals. Cortisol concentrations at both *stress* intervals were significantly higher than those at *rest*.

## 1. Introduction

Fatigue is often associated with photoperiodic/circadian alterations due to sleep deprivation and may lead to the development of affective disorders, psychosomatic diseases, cancer, and other pathologies; alterations in immune and endocrine parameters have been demonstrated among various specialties [1–4]. Special consideration needs to be given to the group of specialties where fatigue is associated with the life-death decision making.

There are few groups of professionals who are subjected to the real-life stressors on a regular basis and whose action may cost lives. Physicians, especially those in training, typically work long hours and are often fatigued due to sleep deprivation; fatigue contributes to the human component of medical errors and, thus, jeopardizes patient safety [5]. Decrease in cognitive function was observed among Ob/Gyn residents and medical students after call [6].

Rollinson et al. demonstrated that visual memory capacity significantly declines in interns across night shift [7]. It is clear that fatigue can affect professional performance of residents. Lapse in concentration and fatigue were the two most common factors contributing to the increase in percutaneous injuries during extended work shifts [8]. Papp et al. found that only 16% of 149 residents from 5 US academic health centers scored within the “normal” range on the Epworth Sleepiness Scale while 84% scored in the range for which clinical intervention is indicated. Residents described multiple adverse effects of sleep loss and fatigue on learning professionalism and task performance [9]. Furthermore, in a prospective, nationwide survey of almost 3000 interns, Barger et al. demonstrated that every extended work shift increased the risk of a motor vehicle crash during the commute from work by 16.2 percent. The authors concluded that extended-duration work shifts, which have been sanctioned by the Accreditation Council for Graduate Medical Education, pose safety hazards for interns [10]. This also poses a hazard for the public. To complicate the situation, current duty hours for surgical residents frequently exceed the proposed ACGME limits. Niederee et al. found that 87% of 1653 surgery residents reported more than 80 duty hours and 45% more than 100 hours per week. More than half of residents felt that their cognitive abilities had been impaired by fatigue [11]. West demonstrated association of fatigue and distress with self-perceived medical errors among medical residents [12].

 The current model of homeostatic interactions suggests that the HPA axis, especially corticosteroids, is the primary driving force for stress-induced secondary immune deficiencies [13, 14]. Additionally, it was found that hydrocortisone and dexamethasone can reactivate the EBV genome in latently infected lymphoblastoid cells [15].

 Conclusively, the body of evidence shows close interaction between those mediators in neuroendocrine-immune network and performance. Moreover, it validates the importance of the systemic analysis of those mediators in response to occupational stresses, which can be quantified objectively and subjectively. We hypothesized that stress response to complex cognitive and noncognitive stressors of the medical training are similar to other occupational stress factors and, as such, can be used as a model for other paradigms (e.g., military service, astronauts) where logistical limitations interfere with the adequate scientific and clinical assessment. The main objective of this study was to evaluate whether fatigue among medical residents is a significant stress factor that is able to affect performance, alter adrenal stress response, and lead to reactivation of the herpes viruses.

## 2. Methods

The experimental design was chosen to evaluate a range of markers of fatigue, including neuroendocrine response and *in vivo* immune state, in healthy humans subjected to fatigue due to sleep deprivation and circadian shifts.

### 2.1. Subjects

A total of 75 residents (45 men, 30 women) (mean age 31.55, SD 5.72 years) from the Mercer University School of Medicine (MUSM) Departments of Internal Medicine (*n* = 22), Pediatrics (*n* = 18), Surgery (*n* = 20), and Family Practice (*n* = 15) completed at least one session. Twenty residents (29%) were in their first year, 19 (28%) in second, 21 (30%) in third, 3 (4%) in fourth, and 6 (9%) were in their fifth; six did not respond.

 Prospective subjects were asked to exclude themselves if they had HBV, HCV, or HIV infection, body organ transplant, known or suspected cancer, used recombinant cytokines, or systemic steroids. This protocol was approved by the MUSM Institutional Review Board. Informed consent was obtained prior to enrollment.

### 2.2. Sample Collection (Intervals and Procedure)

Residents completed the battery of tests at seven intervals: rest, night-float first night, 1 week, 2 weeks, and 4 weeks, 30 hr overnight call start and end of the month, and after call. This paper focuses on three intervals for which saliva and computer values can be compared. For REST intervals, subjects felt rested and had at least 8 hours of sleep prior to testing; *low stress*—following first night of night float; *high stress*—following last night of night float. Samples were collected over 10 months to cover a range of fatigue levels during the various training programs.

### 2.3. Assessment of Fatigue

Residents completed a computerized battery of tests (E-Prime v.1.2., Psychology Tolls Inc., Pittsburgh, PA). The first was a driving simulation; for each shift, nine trials were performed in which reaction time and errors (e.g., brake-pedal push absent stop sign, failure to press for stop sign) were measured. Additionally, subjects completed questionnaires about the work shift, the Epworth Sleepiness Scale, and the Wellness Profile [16, 17].

### 2.4. Evaluation of Immune Status

Immediately after completion of the computer tests, subjects provided saliva samples into Salivettes (Sarstedt AG & Co., Newton, NC, USA) and placed them into a refrigerator. Following collection, samples were centrifuged at 2000 g for 5 min, aliquoted, and frozen at −80°C for the measurement of viral DNA, cortisol, and melatonin.

 Extraction of the viral DNA was performed with the QIAmp DNA Mini Kit (Qiagen, Valencia, CA, USA) from the whole saliva according to the manufacturer recommendations. Presence of DNA for the Herpes simplex virus type-1 (HSV-1), Varicella Zoster virus (VZV), and Epstein-Barr virus (EBV) was analyzed with the semiquantitative real-time PCR technique using SYBR Green chemistry. The following primers were used: HSV-1 (F: CTACAT GGACCCAGTTGTCG, R: CGGTGATTTATACCATGCCA); VZV (F: GCCTCATTTAACGGTTGGTT, R: TCGCGAACTACGTCTTCAAC), and EBV (F: CCGAAGATGAACAGCACAAT, R: TCATCGCTCTCTGGAATTTG). Glyceraldehyde 3-phosphate dehydrogenase (GAPDH; NM_002046.3) and actin-*β* (ACTB; NM_001101) were used as housekeeping genes for the endogenous control (Applied Biosystems, Foster City, CA, USA). All samples were batch-analyzed in duplicate after completion of the study. PCR products were detected on ABI 7300 real-time PCR analyzer (Applied Biosystems, Foster City, CA, USA).

### 2.5. Assessment of the Endocrine Stress Response

Concentrations of cortisol and melatonin were assayed in the saliva with commercial ELISA kits (ALPCO Diagnostics, Salem, NH, USA) and read on the Multiscan MS Plate Reader (Labsystems, Helsinki, Finland) according to the manufacturers' recommendations. All samples were batch-analyzed in duplicate after completion of the study. Sensitivity of the ELISA kits for cortisol and melatonin was 1.0 ng/mL and 0.5 pg/mL, correspondingly.

### 2.6. Statistical Analysis

All analyses were accomplished with SAS version 8.2 (SAS Institute, Cary, NC, USA). Reliability of subjective assessments of fatigue was determined via Cronbach's alpha [18]. Continuous measures were expressed as means ± SD and compared using mixed-model analysis of variance (ANOVA) after determination that distributions confirmed to the requirements of normality and homogeneity. Categorical measures, for example, caffeine use, were expressed as percentages and compared across shifts using Fisher's Exact Chi Square.

## 3. Results

### 3.1. Fatigue Assessment

Nineteen residents (14 men, 5 women) completed the procedure during *rest* and *low-stress* intervals. There were no effects for trial (*P* > 0.45), so mean performance across all nine trials was analyzed. Overall, men (823 ± 782 ms) did not have different reaction times than women (663 ± 1179 ms) and the *rest* (782 ± 634 ms) reaction time was only slightly (*P* = 0.07) faster than the *low-stress* shift reaction time (887 ± 631 ms). The Shift × Gender interaction was significant (*P* = 0.04); this interaction was produced by little shift difference for men (mean_REST_ = 824 ± 723 ms; mean_LOW_ = 782 ± 478 ms) while women were faster at *rest* (663 ± 170 ms) than at *low-stress* (1190 ± 950 ms). For errors, there was a marginally significant shift effect (*P* < 0.08); fewer errors were committed at *rest* (0.37 ± 1.01 *n*/session) than at *low-stress* (1.26 ± 3.30 *n*/session). Only 15 residents (10 men, 5 women) completed the procedure during both *rest* and *high-stress*. Mean reaction time did not differ between shifts nor between genders, nor did the number of errors differ.

**Figure 1:**
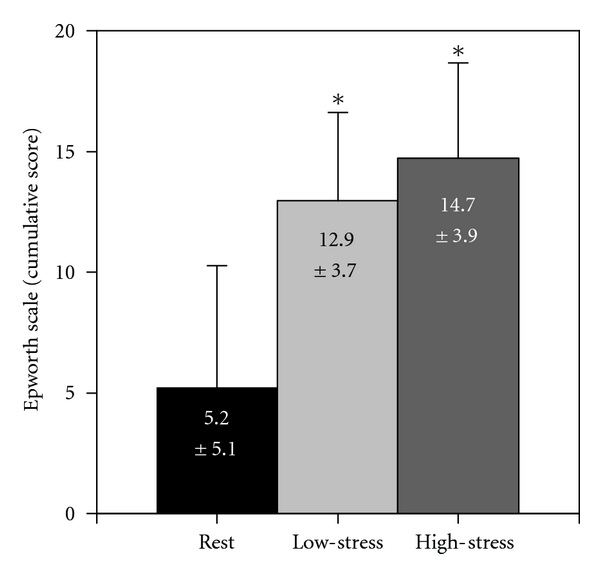
Dynamics in sleepiness among the subjects. Data were analyzed with the Epworth Sleepiness Scale and presented as Mean ± SEM of cumulative score, *n* = 15. *Statistically significant (*P* < 0.05) difference.

The Epworth Sleep Scale evidenced adequate reliability on all three shifts (*α*_REST_ = 0.91, *α*_LOW_ = 0.86, *α*_HIGH_ = 0.88), as did the Wellness scale (*α*_REST_ = 0.72, *α*_LOW_ = 0.72, *α*_HIGH_ = 0.73); the summative score for each scale was therefore used for analysis. Residents reported feeling better (*P* = 0.01) at *rest* (25.00 ± 4.83) than *low-stress* (22.95 ± 4.98) and reported being much less sleepy (*P* < 0.0001) at *rest* than *low-stress*. Furthermore, residents on the *high-stress* shift reported being sleepier (*P* < 0.001) than residents at *rest* interval but did not evidence any difference in Wellness scores (Figure 1). Caffeine use did not differ between any shifts.

### 3.2. Viral Load

**Figure 2:**
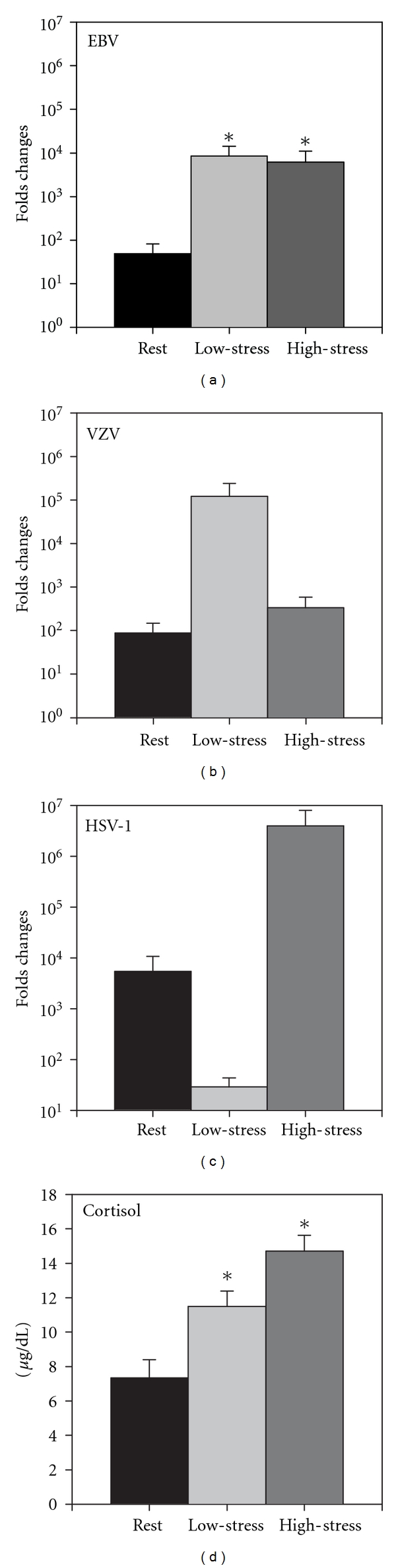
Dynamics of the 3 types herpes virus DNA levels (a–c) and cortisol concentration (d) in saliva of the subjects. Data presented as Mean ± SEM, *n* = 12. *Statistically significant (*P* < 0.05) difference. RQ: relative quantification of SYBR Green qPCR.

Only 15 subjects provided sufficient sample volume to measure viral load for all three viruses at all three intervals. As the first step, log transformation of raw quantification data (RQ) of the viral DNA, which reflect relative changes in the viral load values, detected a Shift × Stress interaction (*P* = 0.015; Figure 2). EBV DNA level increased considerably under both *low*- (log = 2.21 ± 4.41) and *high-stress* (log = 0.75 ± 4.37) conditions, while VZV DNA level (log⁡_LOW_  = 1.44 ± 3.97; log⁡_HIGH_ = 0.33 ± 4.24) increased under stress but to a much lesser extent. DNA level of HSV actually decreased under *low-stress* (log = −0.77 ± 5.18) but increased under *high-stress* (log = 1.47 ± 3.70).

Additionally, for a more comprehensive analysis, actual viral loads were converted to a “Yes/No” response where more than 10-fold increase in viral load was considered as “Yes” response (viral reactivation), while all other (decrease and lower than 10-fold increase) results were considered as “No” response. Overall reactivation was significantly (*P* = 0.02) higher in both *low*- (0.38 ± 0.43) and *high-stress* (mean = 0.27 ± 0.4) than in *rest* (0.00). The three viruses did not differ (*P* = 0.09), and there was no significant interaction (*P* = 0.06).

Insofar as all 3 viruses have different tissue/organ tropism in the host, we combined the categorical results of all 3 by summing the “Yes” and “No” values across the three viruses (Table 1). Thus, a 3 represents a reactivation of all three viruses, a 2 or 1 represents reactivation of two or one of the viruses, respectively, and a 0 represents no reactivation. These values were analyzed via a 3 (stress) repeated measures ANOVA. The stress effect was significant (*P* = 0.02); the total number of reactivations at *rest* (0.26 ± 0.12) was significantly lower than at *low*- (1.6 ± 0.3) and *high-stress* (1.1 ± 0.3). The number of reactivations at *low-stress* was not significantly different from reactions at *high-stress*.

### 3.3. Endocrine Response

Cortisol and melatonin values were analyzed only in 12 subjects for all three levels of stress. There was a significant stress effect (*P* < 0.05). Cortisol concentration at both *low-stress* and *high-stress* intervals was significantly higher compared to *rest* (Figure 2(d)). No significant difference was observed between *low*- and *high-stress* intervals. Additionally, cortisol levels at *rest* and *high-stress* interval were highly correlated (*R* = 0.78, *P* = 0.003), but *low-stress* levels were not correlated with *rest* or with *high-stress*. No significant changes in saliva melatonin level were obtained.

## 4. Discussion

This research project was developed to evaluate the effects of complex stress factors on immune responsiveness, cognitive performance, and fatigue. Medical residency schedule is designed to prepare physicians to adapt to high-stake decision-making stress in real time. In this aspect, residency reflects other occupational stress environments such as active military duty, space exploration, and polar expedition. Chronic exposure to complex cognitive and noncognitive stressors can progress to distress, leads to general fatigue and has been shown to alter immune responsiveness and reactivation of the latent herpes viruses, and affects the subjects' ability to perform their duty [19–23].

In this study, we have detected reactivation of latent herpes viruses that suggests immune suppression. It is not clear if such suppression is generalized or affects specifically antiviral immunity.

As endocrine mediators of the (HPA) axis, glucocorticoids play a primarily suppressive role in regulating immune response [13]. Glucocorticoids affect the immune system by altering leukocyte trafficking and migration of various cell types to areas of inflammation and directly inhibit individual cellular functions [24]. These shifts in leukocyte subpopulations may represent a mechanism to avoid detrimental effects of stress or, alternatively, prepare the immune system for an encounter with antigens and pathogens [25].

 As inflammatory mediators, glucocorticoids inhibit IL-12 production but increase IL-10 production by monocytes and T cells [26]. This drives the immune response towards a Treg cytokine “suppressive” profile and away from a Th1 profile (production of IL-12, IFN-*γ*) that supports cellular immunity essential for viral clearance and, thus, may predispose individuals to herpes virus reactivation [27]. While such viral reactivation was previously well demonstrated [28–31], its underlying mechanisms *in vivo* are not completely understood. Stress, especially chronic “cortisol mediated” as opposed to acute “adrenalin mediated”, is one factor associated with reactivation, and there have been considerable speculation linking stress and the appearance, duration, and intensity of herpes virus infections [32]. Recently, it has been demonstrated that EBV-transformed B-lymphocytes express glucocorticoid receptors and that glucocorticoid hormones, adrenocorticotropic hormone, and corticotropin-releasing factor can reactivate latent EBV *in vitro *[33]. These observations may partially be explained by the recent identification of a glucocorticoid-response element in the BamHI-C fragment of the EBV genome [34]. That also can explain similarity in the pattern of the EBV reactivation with the cortisol saliva level in this study. Cortisol level peaks in the mornings at ~30–45 min after awakening [35, 36]. Samples from our subjects were collected at significantly latter time and often after sleepless nights. As such, it is safe to presume that our data reflect elevated averaged level of the cortisol and, thus, show that even small increase in the concentration of this hormone is able to alter immune responsiveness.

The current set of data does not provide direct explanation of different reactivation pattern among studied viruses. However, we can speculate that differences in the tissue tropism of the herpes viruses, their different sensitivity to endocrine mediators (e.g., cortisol, adrenalin, DHEA, and melatonin), and differences in the organ/tissue involvement in the general adaptation syndrome are significant factors that regulated observed herpes virus reactivation [37, 38].

While significant elevation in the viral load was observed, no clinical manifestations were reported by the participants and correlates with the observation of Cohrs et al. [39]. Individual perception of the stress factors by subjects needs to be considered. Residents are healthy young people who lead an active lifestyle, who realize the importance of a healthy lifestyle (including physical activity and nutrition), and who are very determined to achieve their goals. When the residents were asked whether or not they liked the night-float system, 87% (*N* = 60) responded “yes” and 13% (*N* = 6) responded “no”; 6 did not respond. Such factors may partially counteract the negative side effects of fatigue and sleep deprivation. However, available data confirms that fatigued health practitioners are more likely to make an error, as well as suggests that they are more prompt to develop clinical manifestation of the herpes virus reactivation (shingles, mononucleosis, etc.)

This study has objective limitations. First, busy schedules during this longitudinal protocol produced considerable data loss as many residents were able to complete data collection for only one or two shifts. This loss prevented comparisons among residency programs. Secondly, while saliva collection is a relatively simple procedure, inability to see sample levels and absence of supervision from trained personnel often lead to inadequate collection [40]. Despite the small numbers in the study, the correlates of stress are apparent.

## 5. Conclusions

Fatigue among residents and increased shedding of herpes virus DNA are the major findings of this study. Our subjects have daily close contact with patients in a tertiary care hospital and rotate between intensive care units, operating rooms, the emergency department, inpatient floors, and outpatient clinics. These patients often have a weakened immune system. Increased shedding of viruses by fatigue stressed residents contributes to an unfavorable epidemiological environment. The fact that viral shedding is not perceived by the residents as an illness episode may increase risk of transmission [41, 42]. Moreover, even subclinical reactivation of the latent virus and increase of the viral DNA level could be sufficient to stimulate secretion of the cytokines (e.g., as IFN-*α*, IFN-*γ*, IL-6, IL-12, IL-18, IL-10, and TNF-*α*) with shown psychotropic (e.g., depression, suicidal thoughts) effects (reviewed in: [43–45]). While most of the psychotropic effects were demonstrated for high pharmacological doses, individual response, especially under the stress environment, could be unique and unpredictable.

 Further studies are needed to evaluate the underlying mechanisms of these findings and, ultimately, to develop countermeasures to protect both physicians and their patients.

## Figures and Tables

**Table 1:** Individual dynamics of the DNA levels of the HSV-1, VZV, and EBV in saliva of the subjects. 1, 2, 3—more than 10-fold increase in DNA levels (reactivation) of any 1, 2, or all 3 viruses in saliva; 0—no viral reactivation of any of the viruses. *Statistically significant (*P* < 0.05) difference.

Subject	Rest	Low-stress	High-stress
#1	0	3	0
#2	0	3	1
#3	1	2	1
#4	1	3	0
#5	1	1	3
#6	0	0	1
#7	1	0	0
#8	0	3	1
#9	0	1	1
#10	0	1	0
#11	0	1	0
#12	0	3	3
#13	0	0	2
#14	0	3	0
#15	0	0	3
		*	*
